# Supplementary material for: Gut Microbiota and Oral Contraceptive Use in Women with Polycystic Ovary Syndrome: A Systematic Review
Source: Nutrients. 2024 Oct 4;16(19):3382. doi: 10.3390/nu16193382 (PMC11478613; doi:10.3390/nu16193382)
Supplement: Supplementary file 1 [file nutrients-16-03382-s001.zip › nutrients-3212524-supplementary.pdf]

Table S1. Risk of bias assessment

	**Eyupoglu et al.**	**Garcia-Beltran et al.**	**Tayachew et al.**
**Selection**			
1) Representativeness of the exposed cohort<br>a) Truly representative PCOS in the community * (all subjects or random sampling)<br>b) Somewhat representative of the PCOS of that age in the community * (non-random sampling)<br>c) Selected group<br>d) No description of the derivation of the cohort	0	0	0
2) Sample size<br>a) Justified*<br>b) Non-justified	0	0	0
3) Selection of controls<br>a) Community controls *<br>b) Hospital controls<br>c) No description of the source	0	1	0
**Comparability**			
1) Comparability of cases and controls on the basis of the design or analysis<br>a) Study controls matched for age and BMI *<br>b) Study controls matched for any additional factors such as glucose tolerance, diabetes etc *<br>c) Not reported	2	1	2
**Outcome**			
1) Assessment of microbiota<br>a) Recorded composition with adequate method description *<br>b) No description	1	1	1

2) Same method for ascertainment for cases and controls<br>a) yes *<br>b) no	1	1	1
3) Statistical test<br>a) The statistical test used to describe the data is clearly described and appropriate, and the measurement of the association is presented, including confidence intervals and the probability level (p value) *<br>b) The statistical test is not appropriate, not described or incomplete	1	1	1
